# Whole-genome sequencing reveals insights into the adaptation of French Charolais cattle to Cuban tropical conditions

**DOI:** 10.1186/s12711-020-00597-9

**Published:** 2021-01-04

**Authors:** Lino C. Ramírez-Ayala, Dominique Rocha, Sebas E. Ramos-Onsins, Jordi Leno-Colorado, Mathieu Charles, Olivier Bouchez, Yoel Rodríguez-Valera, Miguel Pérez-Enciso, Yuliaxis Ramayo-Caldas

**Affiliations:** 1 Plant and Animal Genomics, Centre de Recerca en Agrigenòmica (CRAG) CSIC-IRTA-UAB-UB, Campus UAB, Bellaterra, Spain; 2 Université Paris-Saclay, INRAE, Jouy-En-Josas, AgroParisTech, GABI 78350 France; 3 INRAE, SIGENAE, Jouy-En-Josas, 78350 France; 4 INRAE, GeT-PlaGe, Genotoul, Castanet-Tolosan, US 1426 France; 5 Facultad de Ciencias Agropecuarias, Universidad de Granma, Granma, Cuba; 6 Institut Català de Recerca I Estudis Avançats (ICREA), Barcelona, Spain; 7 Animal Breeding and Genetics Program, Institute for Research and Technology in Food and Agriculture (IRTA), Torre Marimon, Caldes De Montbui, 08140 Spain

## Abstract

**Background:**

In the early 20th century, Cuban farmers imported Charolais cattle (CHFR) directly from France. These animals are now known as Chacuba (CHCU) and have become adapted to the rough environmental tropical conditions in Cuba. These conditions include long periods of drought and food shortage with extreme temperatures that European taurine cattle have difficulty coping with.

**Results:**

In this study, we used whole-genome sequence data from 12 CHCU individuals together with 60 whole-genome sequences from six additional taurine, indicus and crossed breeds to estimate the genetic diversity, structure and accurate ancestral origin of the CHCU animals. Although CHCU animals are assumed to form a closed population, the results of our admixture analysis indicate a limited introgression of *Bos indicus*. We used the extended haplotype homozygosity (EHH) approach to identify regions in the genome that may have had an important role in the adaptation of CHCU to tropical conditions. Putative selection events occurred in genomic regions with a high proportion of *Bos indicus*, but they were not sufficient to explain adaptation of CHCU to tropical conditions by *Bos indicus* introgression only. EHH suggested signals of potential adaptation in genomic windows that include genes of taurine origin involved in thermogenesis (*ATP9A*, *GABBR1*, *PGR*, *PTPN1* and *UCP1*) and hair development (*CCHCR1* and *CDSN)*. Within these genes, we identified single nucleotide polymorphisms (SNPs) that may have a functional impact and contribute to some of the observed phenotypic differences between CHCU and CHFR animals.

**Conclusions:**

Whole-genome data confirm that CHCU cattle are closely related to Charolais from France (CHFR) and Canada, but also reveal a limited introgression of *Bos indicus* genes in CHCU. We observed possible signals of recent adaptation to tropical conditions between CHCU and CHFR founder populations, which were largely independent of the *Bos indicus* introgression. Finally, we report candidate genes and variants that may have a functional impact and explain some of the phenotypic differences observed between CHCU and CHFR cattle.

## Background

Climate change and global warming are among the main challenges currently faced by Agriculture and Livestock husbandry. In this scenario, it is fundamental to investigate the mechanisms that allow animals to adapt to high temperatures. The hot temperature conditions of tropical climates today might resemble those that animals raised outdoors under temperate climates, such as most beef cattle and small ruminants, will face in the future. Therefore, animals that currently live in hot climates and that have a European or temperate climate origin can provide clues into the genetic mechanisms underlying adaptation to increasing temperatures [1].

Cattle breeds can be divided into temperate taurine breeds *Bos taurus*, of European origin, and Indian zebu breeds *Bos indicus*, which diverged ~ 250,000 years ago [2]. *B. indicus* breeds produce less meat and of lower quality but are more adapted to heat and parasites than taurine breeds. For that reason, they were imported to tropical American regions starting in the mid-19^th^ century [3, 4]. Under these tropical climates, they have largely replaced the primigenious cattle that were imported by the first Spanish and Portuguese settlers [5]. Numerous hybrid populations between *B. taurus* and *B. indicus*, such as the Brangus, Texas Longhorn, Santa Gertrudis, among others, also coexist with pure *B. indicus* breeds. These mixed breeds exhibit a good resistance to parasites and heat, and also produce carcasses of much higher quality than pure *B. indicus* breeds.

At the beginning of the 20th century, Cuban farmers imported Charolais animals from France, which have resulted in a population of cattle now known as ‘Chacuba’ (CHCU). This population has adapted to the breeding conditions in the Cuban tropical environment in ~ 20 generations of breeding. In this short period of time, several clear phenotypic differences between the original French Charolais (CHFR) and its Cuban counterpart have appeared. CHCU cattle are smaller than CHFR, with weights of 34 vs. 46 kg at birth and 290 vs. 493 kg for heifer’s weight at 18 months, respectively [6, 7]. Also, CHCU cattle are hairless and their carcasses have a lower grade and higher fat content than CHFR [7]. Although the CHCU cattle are thought to form a closed population with no records of interbreeding, Ribas [8] reported the presence of a specific *B. indicus* blood group allele (U’_1_), at a very low frequency. More recently, Rodriguez-Valera et al. [6] used the Illumina Bovine 50 k single nucleotide polymorphism (SNP) BeadChip to investigate the genetic structure and putative ancestral origin of this population and showed that CHCU clusters with the taurine breeds. Nevertheless, in spite of the short period of time since the importation of Charolais animals from France, a marked differentiation (fixation index (*F*_ST_) = 0.049) is observed between CHFR and CHCU cattle. Genetic and phenotypic differentiation can be caused by genetic drift, i.e., as a consequence of the importation of a small number of individuals and by selection of beneficial variants that promote adaptation to the tropical environment. A number of statistics have been proposed to distinguish changes produced by genetic drift from those due to adaptation (e.g., [9–11]).

Because SNP array genotype data are biased and have a low resolution, for our study, we obtained whole-genome sequence data from 72 animals, including 12 CHCU animals, to provide an unbiased estimation of the population structure and to fine-map regions that could have played a role in the adaptation to tropical conditions of the Chacuba population.

## Methods

### Samples

Seventy-two whole-genome sequences from taurine, indicine and crossbred cattle were analyzed. CHCU cattle have been maintained under pedigree control at the “Manuel Fajardo” genetic center that is located in Jiguani (Granma Province) with a current census of ~ 700 animals. Therefore, the genetic relationship between animals can be accurately tracked and we used this information to select 12 unrelated CHCU animals that were sequenced in this work. We also used 15 French Charolais (CHFR) [12], six Limousine (LIMS) from France [13] and sequences from 39 additional individuals that were downloaded from the sequence read archive (SRA) database [see Additional file 1: Table S1]: 15 Canadian Charolais (CHCA), five Limousine from Canada (LIMS), five Brangus (BRG), 10 Brahman (BRM) and four Texas Longhorn (TXL). Brahman is a pure *B. indicus* breed whereas BRG and TXL are admixed breeds between *B. indicus* and *B. taurus* cattle. Data on the French and Canadian LIMS individuals were merged in the analyses reported here.

### Bioinformatic analysis

All the sequences were mapped against the bovine reference assembly (UMD3.1.1) using the Burrows-Wheeler aligner (BWA) v. 0.7.12-r1039 software [14]. PCR duplicates were removed using the Picard MarkDuplicates (v2.18.9) program and realigned around InDels with the GATK IndelRealigner tool [15]. For each individual, SNP calling was done with the SAMtools mpileup and bcftools call (v. 0.1.19-96b5f2294a) tools with the following parameters: minimum and maximum depths between 5 × and twice the average sample’s depth; a minimum SNP quality of 10; and a minimum mapping quality and minimum base quality of 20. Next, we merged individual gVCF files into a multi-individual VCF file, with all the SNPs from the 72 samples. For this purpose, we followed a two-step approach as detailed in [16], using a pipeline available at https://github.com/miguelperezenciso/NGSpipeline. In brief, to identify whether a position is equal to that in the reference genome, polymorphic or missing, first we generated a fasta file from the gVCF file for each individual and generated a multi-individual VCF file by using the individual file. Once the multiple sample file was obtained, SNPs with more than 20% missing data across samples and populations were removed. Finally, we imputed the missing genotypes and inferred phases with the Beagle 4.1 software [17].

Genetic variants that alter transcription factor binding sites (TFBS) were predicted with a custom script using the TFBS models from the JASPAR (JASPAR CORE 2018 collection, [18]), HOCOMOCO (version v10, [19] and TRANSFAC (version v3.2 public, [20] databases. These databases contain a curated set of TFBS models represented as position weight matrices (PWM), which are derived from published collections of experimentally defined eukaryotic TFBS. Only vertebrate PWM were downloaded and used in our study. Finally, we identified microRNA binding sites by using the TargetScan (release 7.2) software [21].

### Population genomics

We estimated Watterson’s nucleotide variability (θ) [22] and differentiation values (*F*_ST_, [23] between populations with the mstatspop (v.0.1beta, https://github.com/CRAGENOMICA/mstatspop) software in consecutive non-overlapping 30-kb windows. This software implements algorithms that allow for missing data [24]. For the remaining analyses, we imputed missing genotypes with Beagle 4.1 [17]. The EHH-derived statistics (Rsb, and iHs) [25] were computed between CHCU and CHFR for each SNP (https://github.com/CRAGENOMICA/Tang_Rsb). As putative selection events, we retained the windows with a ‘permutation p-value’ lower than 0.05 among the 2000 windows that contained the SNPs with the largest Rsb value. The ‘permutation p-value’ was obtained by randomly shuffling the CHCU and CHFR samples and running the Rsb algorithm along the whole genome. The process was repeated 100 times and we computed the number of times the observed Rsb statistics was larger than the values obtained from permutation, and obtained a ‘permutation p-value’ for each SNP. This process aims at correcting for different levels of disequilibrium along the genome that may locally inflate Rsb values. In a second step, we performed the same procedure but this time between CHFR and CHCA, to exclude the Rsb intervals that were common between CHFR and CHCA and not specific to CHCU. Therefore, we focused only on the Rsb intervals with signals exclusively from CHCU.

### Admixture

The software ADMIXTURE v1.3.0 [26] was run in an unsupervised manner with a number of clusters K = 2 using CHCU, BRM, CHFR and CHCA genotypes. We chose K = 2 because we were only interested in ascertaining *B. indicus* introgression in CHCU. Nevertheless, K = 2 was also the value that resulted in the lowest cross-validation error. The program was run either by including all the SNPs or with pruned data from which SNPs in strong disequilibrium were removed, but the results were identical. To get a more precise map of potential admixture, we used the ELAI software [27], which is a partially supervised algorithm that requires data from the putative founder populations (CHFR and BRM) and the potentially admixed population (CHCU). Therefore, ELAI was run using CHCU, CHFR and BRM genotypes only. ELAI implements a two-layer hidden Markov model and was run by setting the recommended default parameters, which included removal of SNPs with a minimum allele frequency (MAF) lower than 0.01.

## Results

### Population structure and impact of *B. indicus* introgression on CHCU

Average read depth across breeds varied between 8.7 (LIMS) and 12.5 (CHCU) (see Table 1). We found 42,144,809 SNPs among which 14,929,949 were specific to the pure *B. indicus* breed (BRM), 6,839,436 to the taurine breeds and 1,176,249 to CHCU. As expected [28], *B. indicus* samples were more variable than *B. taurus* samples, i.e. the nucleotide variability was equal to 0.0035 per bp for BRM but was two times lower for LIMS and European Charolais (Table 1). The hybrid breeds TXL and BRG had an intermediate level of genome diversity, i.e. between those of the *B. indicus* and *B. taurus* breeds, and CHCU had a level of genome diversity similar to that of TXL (Table 1). Thus, it is interesting to note that although the number of founders of the CHCU population is small, it has a higher nucleotide variability than its ancestral CHFR population.

**Table 1:** Description of the breeds analyzed, shared and breed-exclusive SNPs

Breed	Country	Number of samples	Wattersons variability	% missing	Mean depth	CHCU-exclusive SNPs	Shared SNPs with CHCU	Shared SNPs with CHFR	Shared SNPs with CHCA	Shared SNPs with LIMS	**Shared SNPs with TXL**	**Shared SNPs with BRG**
CHCU	CU	12	0.0021	0.03	12.5	1,176,249	**–**					
CHFR	FR	15	0.0015	0.06	11.4	582,972	10,110,342	**–**				
CHCA	CA	15	0.0018	0.13	10.4	737,160	10,667,212	10,847,599	**–**			
LIMS	FR/CA	11	0.0016	0.21	8.7	695,594	9,429,688	9,796,472	9,961,440	**–**		
TXL	US	4	0.0023	0.08	10.3	576,849	8,243,412	7,639,145	8,085,077	7,370,881	**–**	
BRG	US	5	0.0028	0.05	11.5	842,771	9,514,147	6,854,966	9,033,938	7,934,515	7,655,123	**–**
BRM	US/AU	10	0.0035	0.11	10.8	14,929,949	11,442,083	8,653,635	10,134,007	8,267,282	9,169,187	12,519,875

**Fig. 1:**
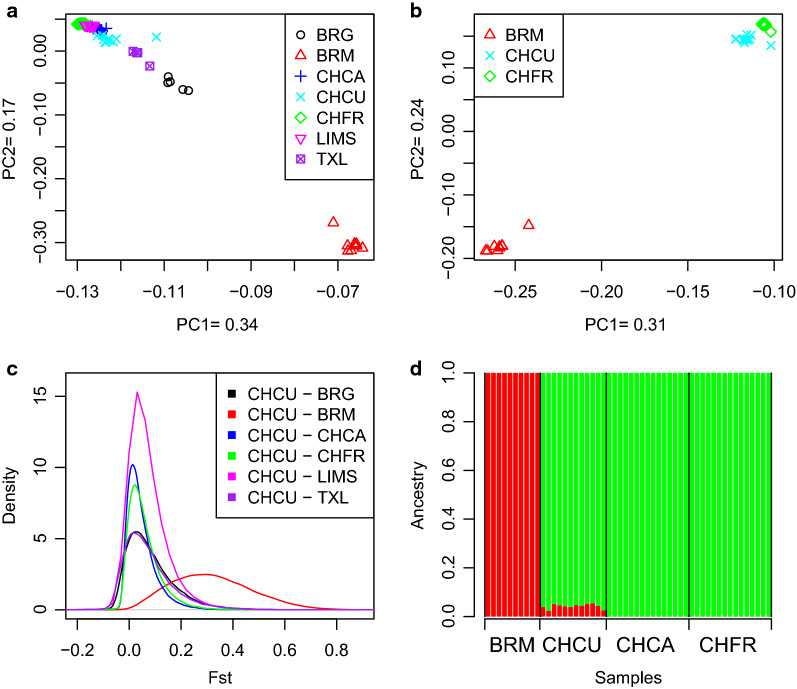
Population structure. (**a**) Principal component analysis using all samples. Individuals are grouped into *Bos taurus, Bos indicus* and Hybrid clusters. Black: Brangus, red: Brahman, blue: Canadian Charolais, cyan: Cuban Charolais, green: French Charolais, magenta: Limousin, purple: Texas Longhorn (**b**) Principal component analysis using all samples. Red: Brahman, cyan: Cuban Charolais, green: French Charolais (**c**) *F*_ST_ between CHCU and the other breeds. (**d**) Results of admixture analyses with two ancestral populations (K = 2) Red: Brahman (*Bos indicus*) and green: French and Canadian Charolais (*Bos taurus*)

In line with previous observations [29, 30], the principal component analysis (PCA) plot shows a clear separation between indicine and taurine breeds, with the latter breeds being tightly clustered (Fig. 1a). As expected, animals sampled from the admixed breeds BRG and TXL, are positioned towards the cluster of indicine breeds but much closer to the taurine than to the indicine clusters, because their proportion of indicine genome is less than 50% or even 11% in the case of TXL breed [31]. Regarding the CHCU individuals, they are positioned near the LIMS and Canadian Charolais, but separated from the original French Charolais. Since one CHCU individual appeared to be an outlier, we inspected its genotype heterozygosity patterns (11%), but we could not find any anomalous deviation. Moreover, this individual does not appear as an outlier when only BRM, CHCU and CHFR are represented in a separate PCA plot (Fig. 1b). In terms of *F*_ST_, the closest populations to CHCU were the Canadian (0.04) and French Charolais (0.05) (Table 1). The plot of *F*_ST_ values across 1-Mb windows between CHCU and the taurine breeds shows a modal value near zero, whereas that between CHCU and the indicine breeds shows that they are clearly distinct (Fig. 1c).

In previous work based on the Illumina 50 k SNP Chip genotype data, we reported a putative introgression of *B. indicus* into CHCU and although it was small [6], it could explain why CHCU and CHFR are clearly separated, in spite of the short period since the original importation of Charolais from France. Here, we confirm this introgression with a better resolution using whole-genome data. As shown in Fig. 1d, an unsupervised Admixture analysis with K = 2 clusters clearly separates the taurine CHCA and CHFR animals from the pure *B. indicus* BRM breed, whereas it reveals a small introgression of *B. indicus* in CHCU. This is also reflected when only the BRM, CHFR and CHCU breeds are represented in the PCA plot (Fig. 1b).

**Fig. 2:**
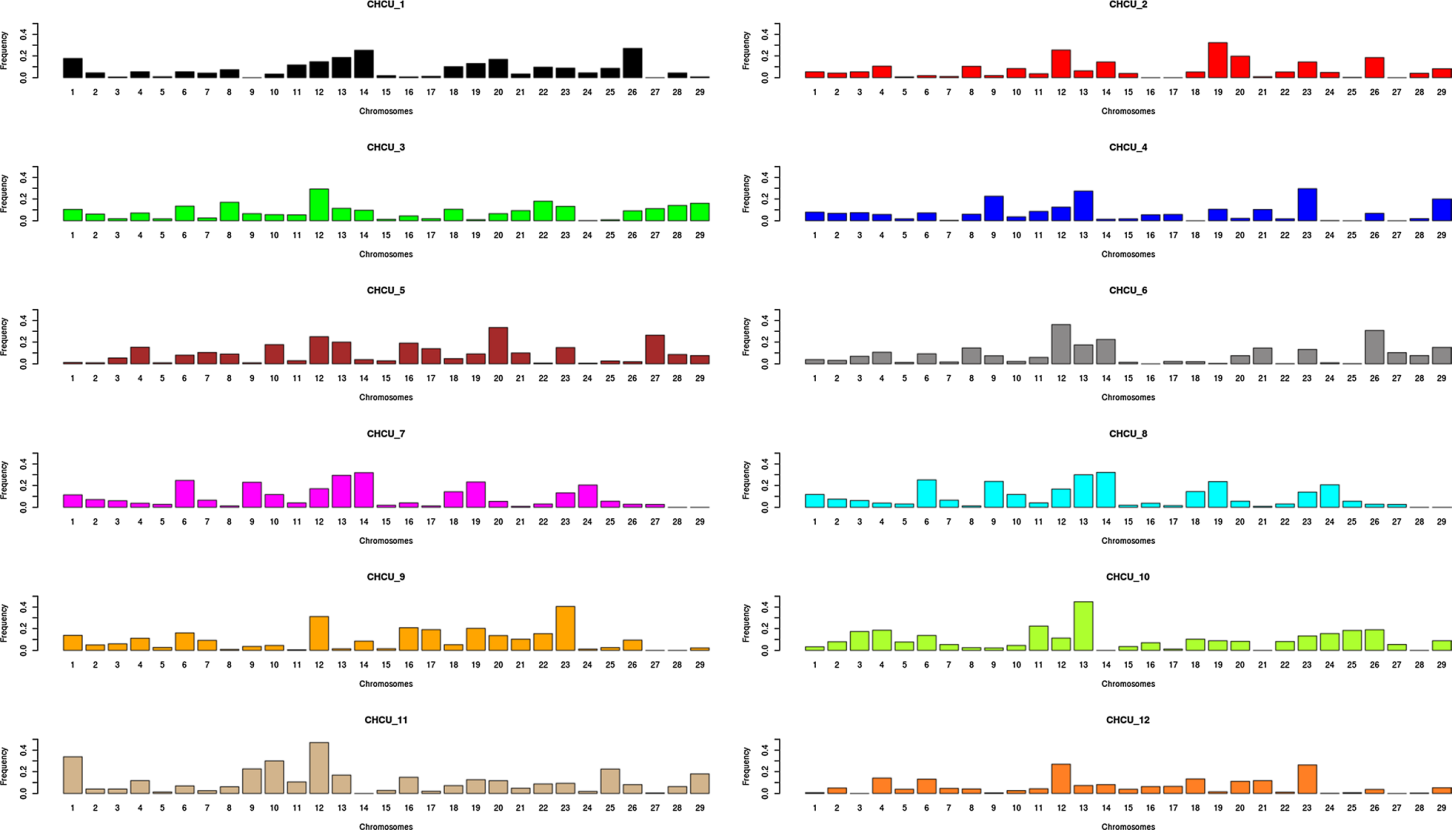
Frequency of indicus component per CHCU sample and chromosome inferred from analysis with the ELAI software

To evaluate more precisely the extent and impact of *B. indicus* introgression into CHCU, we ran the ELAI software [27], which provides a map, for each hybrid individual, showing the probability for each SNP to descend from one of the two putative founder populations. The results show that the *B. indicus* introgression is not homogeneous, neither across individuals nor across chromosomes (Fig. 2) and [see Additional file 2: Figure S1]. For instance, on average, *B. taurus* (BTA) autosomes BTA12, 13 and 23 had the highest proportions of *B. indicus* introgression (i.e. 24, 19 and 18%, respectively) across all animals. On a per individual basis, the highest proportion of *B. indicus* introgression was found for the CHCU11 individual, i.e. 47% on BTA12, followed by CHCU10, i.e. 45% on BTA13. In contrast, on average the lowest proportion of *B. indicus* introgression was found for individuals CHCU12 (6.5%), CHCU4 (7.4%) and CHCU2 (7.5%) and for chromosomes BTA5 (2.4%), BTA15 (2.7%) and BTA28 (3.9%). Additional file 3: Figure S2 [see Additional file 3: Figure S2] shows the correlation between the proportion of *B. indicus* genome in CHCU and the *F*_ST_ value between CHCU and CHFR across windows. A higher proportion of *B. indicus* was moderately correlated (0.19) with a greater differentiation between the Charolais populations.

### Putative selective sweeps between Cuban and French Charolais

**Fig. 3:**
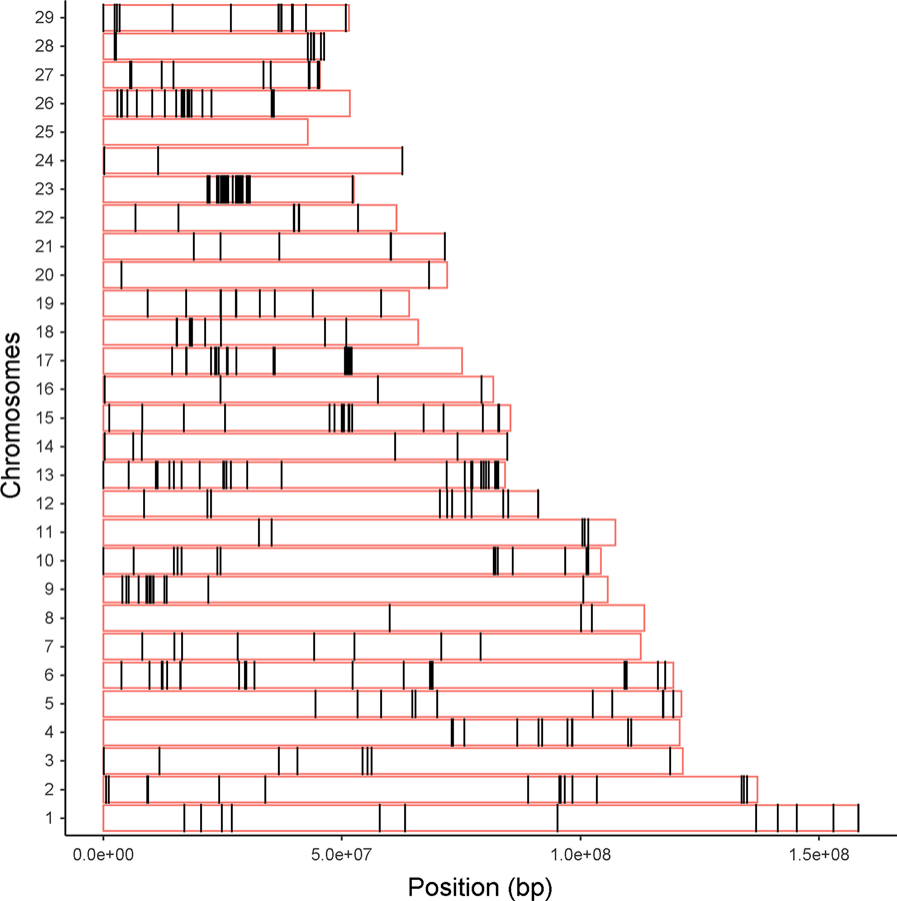
Extended haplotype homozygosity (EHH) intervals per chromosome. The x-axis represents the chromosome positions and the y-axis the chromosome number

**Fig. 4:**
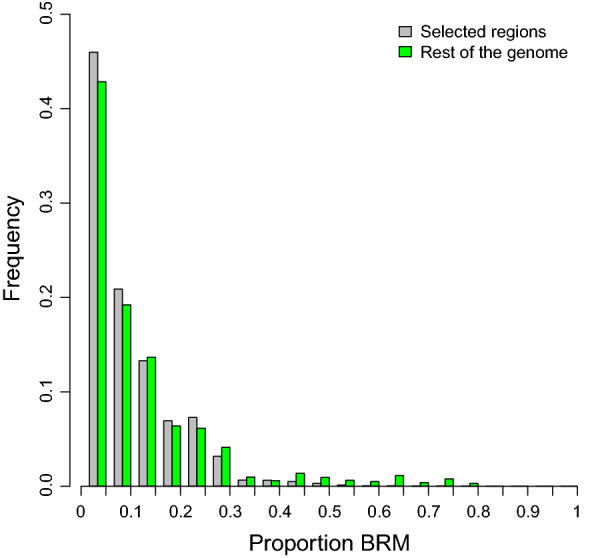
Percentage of BRM in windows containing putative selection events vs. the other windows, inferred from analysis with the ELAI software

Among the 2000 windows with the largest positive Rsb value, we selected the 407 windows with a ‘permutation p-value’ lower than 0.05 and exclusive of CHCU (Fig. 3), i.e., we excluded the significant intervals observed in the comparison between CHFR and CHCA, since introgression and adaptation to a hot climate are not expected to have played a role in any of the founder populations [see Additional file 4: Table S2]. In addition, we focused on positive Rsb values only, since we are interested in regions where the putative selective pressure is specific to the Cuban population, i.e., where the disequilibrium is larger in CHCU than in CHFR. Figure 4 shows the distribution of the percentage of indicus introgression for the putative selective windows and the rest of the genome. The average percentages of Brahman introgression were 0.22 and 0.17% in the selected and control windows, respectively. The difference, although small, was significant (P < 5e-09) according to a Wilcoxon rank test.

We identified 243 genes within the 407 selected windows, including five (*ATP9A*, *GABBR1*, *PGR*, *PTPN1* and *UCP1*) involved in thermotolerance and two (*CCHCR1* and *CDSN*) involved in hair development (Table 2). The use of whole-genome data, allowed us to also pinpoint genetic variants within these genes that may have a functional impact and thus may explain some of the phenotypic differences that exist between CHCU and CHFR animals. For example, 1253 SNPs were detected within the *ATP9A* gene, which is associated with heat tolerance in pigs [32]. Among these SNPs, one (rs207874965) is located within the 3′UTR, five SNPs have the potential to alter splicing, and 22 SNPs are in the upstream region, including 20 that modify TFBS. We found 251 SNPs within the *GABBR1* (*gamma*-*aminobutyric acid B receptor 1*) gene, which, if inactivated, induces hypothermia in mice [33], 19 of these SNPs are within the 3′UTR and two SNPs (rs110080552 and rs210443447) have the potential to impact splicing. In addition, 227 of the 229 SNPs located in the upstream region of *GABBR1* can potentially modify the binding site of transcription factors (see Additional file 5: Table S3). Interestingly, nine of these potential regulatory SNPs are fixed for the alternative allele in CHCU. We found 1182 SNPs in the *PGR* gene, which encodes the progesterone receptor and is associated with thermotolerance in cattle [34]. Among these 1182 SNPs, two can impact splicing and 16 are located in the upstream region and can potentially modify TFBS (see Additional file 5: Table S3).

**Table 2:** Selected candidate genes located within the putative selective intervals

Genome position	Gene name	Size (bp)	Number of SNPs	Function	Reference
13:79263518-79324185	*PTPN1*	60,667	382	Heat tolerance	Klaman et al. [35])
13:80167668-80262763	*ATP9A*	95,095	1253	Heat tolerance	Kim et al. [32])
15:8104485-8222755	*PGR*	118,270	1182	Heat tolerance	Tsubota et al. [41]
17:17467450-17473822	*UCP1*	6372	118	Heat tolerance	Charkoudian and Stachenfeld [38]
23:27779849-27791241	*CCHCR1*	11,392	133	Hair development	Oka et al. [36]
23:27808496-27812710	*CDSN*	4214	466	Hair development	Leclerc et al. [48]
23:28775534-28803895	*GABBR1*	28,361	251	Heat tolerance	Haller et al. [33]

Within the *PTPN1* (*protein*-*tyrosine phosphatase 1B*) gene, we detected 382 SNPs including three located in the 3′UTR and seven located in the upstream region that can potentially alter TFBS. Inactivation of *PTPN1* in mice results in an increase in adaptative thermogenesis [35] and therefore some of these candidate regulatory SNPs might explain the difference in heat tolerance between CHFR and CHCU. Finally, among the candidate genes involved in thermogenesis, we found two deleterious missense variations (rs443726914 and rs715309385) within the *UCP1* gene and 77 upstream SNPs, including four that are fixed (rs438305189, rs211174809, rs209939359 and rs211622720) for the alternative alleles in CHCU (see Additional file 5: Table S3). Interestingly, these four upstream SNPs can potentially alter the binding sites of several transcription factors.

Regarding hair development, 133 SNPs were found within the *CCHCR1* gene, which is involved in hair loss [36]. Three SNPs (rs110552603, rs207611773 and rs381805999) are located within the 3′UTR and two SNPs (rs17871433 and rs377855638) can potentially impact splicing. In addition, we found ten missense SNPs and, in the upstream region, seven SNPs, which all change TFBS and one of them (rs109692098) is fixed for the alternative allele in CHCU. Another candidate gene involved in hair development is the *corneodesmosin* gene (*CDSN*), which contained 466 SNPs within our bovine samples, among which one SNP (rs462034580) can potentially impact splicing of this gene, 194 SNPs are located in the upstream region, and three are missense deleterious SNPs (rs209222317, rs434552200 and rs479537418). Interestingly, one of the *CDSN* deleterious mutations (rs434552200) is nearly fixed for the alternative allele in CHCU, with a frequency of 0.994. Further experimental analysis of the impact of these variants is needed to determine whether they are involved in some of the phenotypic differences between CHCU and CHFR.

## Discussion

Creole cattle refer to the descendants of the first European animals that have adapted to local tropical conditions on the American continent. However, as Burgos-Paz et al. [37] showed for ‘creole’ pigs, the origin of these animals is usually mixed and, usually there is little trace of the original founders However, in very few cases, the origin of extant animals can be tracked accurately and, for that reason, CHCU is a unique population for which pedigree records have been maintained over most of the time and isolation has been, in principle, guaranteed.

In spite of this assumed isolation, our findings confirm that CHCU was crossed with *B. indicus* animals, as previously suggested by Rodriguez-Valera et al. [6] based on SNP array data. In our study, we estimated that the percentage of the CHCU genome originating from *B. indicus* is in the order of 4 to 8%, depending on the method used or the individual considered (Figs. 1d and 2, and (see Additional file 2: Figure S1)). The proportion of *B. indicus* component varied largely, both across chromosomes and individuals (Fig. 2). If the *B. indicus* introgression is assumed to be a recent and sporadic event, it explains this imbalance across chromosomes and individuals, which will be smoothed out in the future generations as recombination events increase, and the correlation of the proportion of the *B. indicus* alleles between individuals across windows is expected to increase with time. We found a positive low correlation between proportion of *B. indicus* and *F*_ST_ (see Additional file 3: Figure S2), which suggests that the divergence between founder (CHFR) and derived (CHCU) populations is probably not due to *B. indicus* introgression only.

The genetic differentiation in CHCU may also result from the strong bottleneck that occurred during the importation process, since it is likely that only a few haplotypes were introduced in Cuba. This differentiation might have increased due to genetic drift if the effective size of this population remained low during the following generations. Founder effect and genetic drift are likely the main causes of the whole genomic differentiation at the non-functional positions of the genome, together with *B. indicus* introgression, since an increase of exclusive variability also increases population differentiation. Nevertheless, selection of genetically beneficial variants can also shape the local genetic differentiation at specific regions, as well as the phenotypic patterns of this breed. The statistics that we used should correct for demographic effects, and should allow us to detect unusual patterns that are compatible with positive selection.

The *B. indicus* component had a measurable impact on increasing nucleotide variability in CHCU compared to that in the European breed, more than offsetting the effect of the founder bottleneck (Table 1). It is tempting to hypothesize that the detected *B. indicus* introgression is related to the adaptation of CHCU. If this was the case, genomic regions with a high proportion of *B. indicus* should be enriched in signatures of selection. We did find a significant excess of *B. indicus* component within selective windows (Fig. 4), but it was too small to explain all the adaptive events detected. Although the *B. indicus* genomic component is associated with adaptation (Fig. 4), the alleles of *B. indicus* origin cannot be considered as the main drivers of selection. This leads us to hypothesize that, likely, most of the adaptation events that have occurred in CHCU are due to changes in allele frequencies that were already present in the French Charolais, i.e., soft sweeps.

Our results confirmed 17 of the 104 genomic regions reported by Rodriguez-Valera et al. [6]. The difference in the number of identified genomic intervals between these two studies can be partly explained by the fact that Rodriguez-Valera et al. [6] annotated both positive and negative extreme Rsb values and did not apply a second filter based on the permutated *p* value. In our study, we focused on positive and significant (permutated p-value < 0.05) genomic intervals, since our aim was to detect regions in which the putative selective pressure is specific to the Cuban population. Moreover, the detection of putative signatures of selection in [6] was based on SNP chip data and, consequently, the sizes of the genomic intervals were much larger (~ 700 kb vs. 30 kb here). Therefore, our results provide a better resolution and accurate identification of putative signatures of selection between CHCU, CHFR and CHCA, which in turn facilitates the identification of genetic variants within candidate genes related to the adaptation to tropical conditions.

Within the selective windows, we found seven genes related to thermotolerance and hair development (Table 2), which are both key traits in the adaptation to tropical conditions and might explain some of the phenotypic differences between the CHFR and CHCU breeds. Regarding thermotolerance, the genes *ATP9A*, *GABBR1*, *PGR*, *PTPN1* and *UCP1* were putatively under selection. Previously, Dikmen et al. [34] reported an association between a SNP in *PGR* and rectal temperature in US Holstein lactating cows exposed to heat stress. This SNP (rs109506766) is an intronic *G/C* SNP and, interestingly, the frequency of the *G* allele differs between CHCU and CHFR (0.38 versus 0.53). We also found several SNPs in *PGR* that had large differences in allele frequency between CHCU and CHFR and that might have a potential functional impact. For example, 16 of these SNPs can alter the binding sites of transcription factors, and interestingly, seven of these candidate regulatory variants modify the binding sites of heat shock factors.

We also found one deleterious missense variant (r42676011) in the codon for an arginine amino-acid within the ligand-binding domain of the *progesterone receptor* (*PGR)* gene, which is conserved among 70 eutherian mammals. Several studies have shown that progesterone has a vasoconstrictive effect, which reduces the cutaneous blood flow when the temperature of the skin increases, and ultimately decreases heat dissipation (*e.g*. [38]. Moreover, glucocorticoids induce heat resistance in mammalian cells, whereas progesterone, a glucocorticoid antagonist, inhibits the development of this resistance [39]. These findings suggest that *PGR* might play an important physiological role in reducing body heat loss.

The mitochondrial *uncoupling protein 1* (*UCP1*) gene is predominantly expressed in brown adipose tissue and plays major roles in regulating body temperature, metabolic rate and controlling energy expenditure via both non-shivering thermogenesis and diet-induced thermogenesis [40]. *UCP1* is essential for maintaining body temperature in non-cold conditions [41]. Variants in the bovine *UCP1* have been found to be associated with milk yield, milk fat percentage and milk protein percentage [42], but to our knowledge not to thermotolerance. We can hypothesize that genetic variations within *UCP1* might partly explain the heat tolerance of the CHCU cattle. These variants should have a negative impact on *UCP1* by reducing its activity or expression, and thus it would be interesting to perform an experimental confirmation.

*CCHCR1* is involved in hair development and encodes the coiled-coil alpha-helical rod protein 1, which is expressed in basal keratinocytes [43]. Its exact function remains unknown, but it has been shown to play a wide variety of roles in steroidogenesis, proliferation, differentiation and cytoskeletal organization [44, 45]. Interestingly, Oka et al. [36] have identified a missense variant in *CCHCR1* that is associated with alopecia areata, an auto-immune disease affecting the hair follicle. We found several functional candidate variants within *CCHCR1* that had large differences in allele frequency between CHCU and CHFR. For example, two of these SNPs can potentially disrupt the binding sites of heat shock factors. As previously mentioned, the *corneodesmosin* (*CDSN)* gene is also related to hair development. Mutations in the human *CDSN* are associated with hypotrichosis simplex, a scalp-specific hair loss [46] and mice having undergone targeted inactivation of *Cdsn* showed rapid hair loss, which confirms the essential role of *Cdsn* for maintaining the architecture of the hair follicle [47, 48].

## Conclusions

In conclusion, analysis of whole-genome data confirms that the CHCU animals included in this study are closely related to Charolais cattle from France and Canada, but also reveal a limited introgression of *B. indicus* in CHCU. We observed signals of recent adaptation to tropical conditions between CHCU and CHFR founder populations, which are largely independent of the *B. indicus* introgression, which suggests that most of the selection events are caused by soft sweeps. Some of the identified regions harbor genes that are involved in thermogenesis (e.g., *ATP9A*, *GABBR1*, *PGR*, *PTPN1* and *UCP1*) and hair development (*CCHCR1* and *CDSN*). We also report the presence of SNPs within these genes that can have a functional impact and might explain some of the phenotypic differences observed between CHCU and CHFR animals. Future experimental work is needed to evaluate the role of these a priori relevant genes in CHCU adaptation.

## Supplementary information

**Additional file 1: Table S1.** Description of the samples included in this study.

**Additional file 2: Figure S1.** Proportion of *indicus* per CHCU samples.

**Additional file 3: Figure S2.** Relationship between proportion of *indicus* in CHCU and *F*_ST_ CHCU-CHFR, each dot corresponds to the *F*_ST_ value of a 30-kb window.

**Additional file 4: Table S2.** Description of the intervals identified as selective sweeps between Cuban and French Charolais.

**Additional file 5: Table S3.** Putative regulatory SNPs (rSNPs) located upstream of interesting genes.
