# Supplementary figures and images for: Whole-genome sequencing reveals insights into the adaptation of French Charolais cattle to Cuban tropical conditions
Source: Genet Sel Evol. 2021 Jan 4;53:3. doi: 10.1186/s12711-020-00597-9 (PMC7784321; doi:10.1186/s12711-020-00597-9)

**CHCU\_1**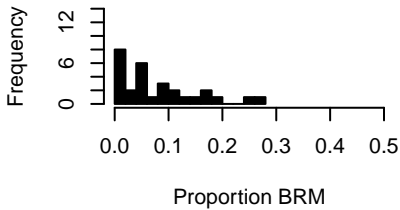**CHCU\_2**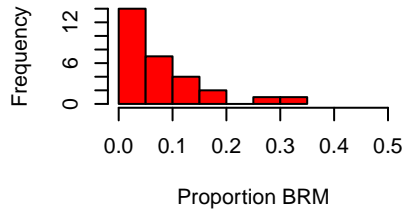**CHCU\_3**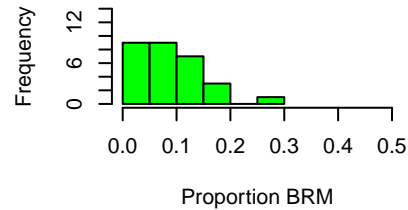**CHCU\_4**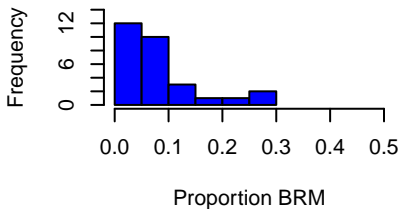**CHCU\_5**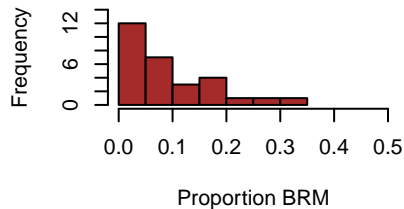**CHCU\_6**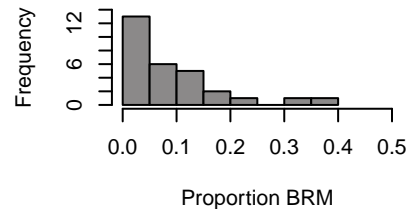**CHCU\_7**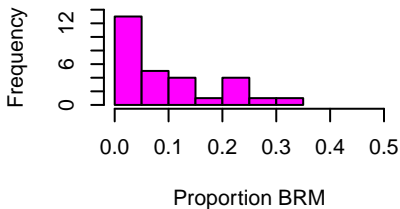**CHCU\_8**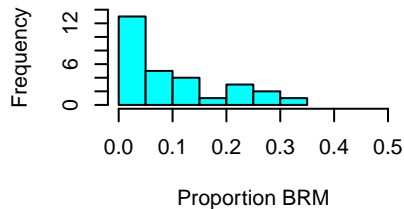**CHCU\_9**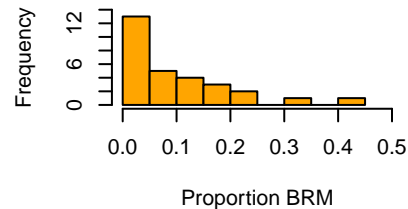**CHCU\_10**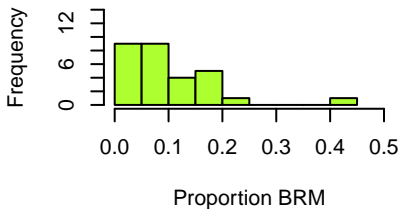**CHCU\_11**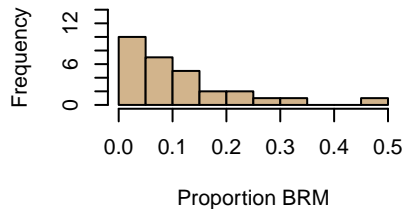**CHCU\_12**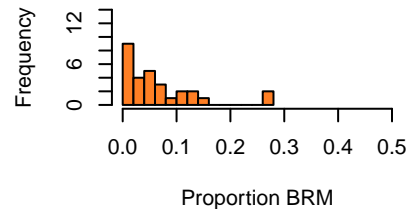

Supplement: Supplementary file 2 — Additional file 2: Figure S1. Proportion of indicus per CHCU samples. [file 12711_2020_597_MOESM2_ESM.pdf]

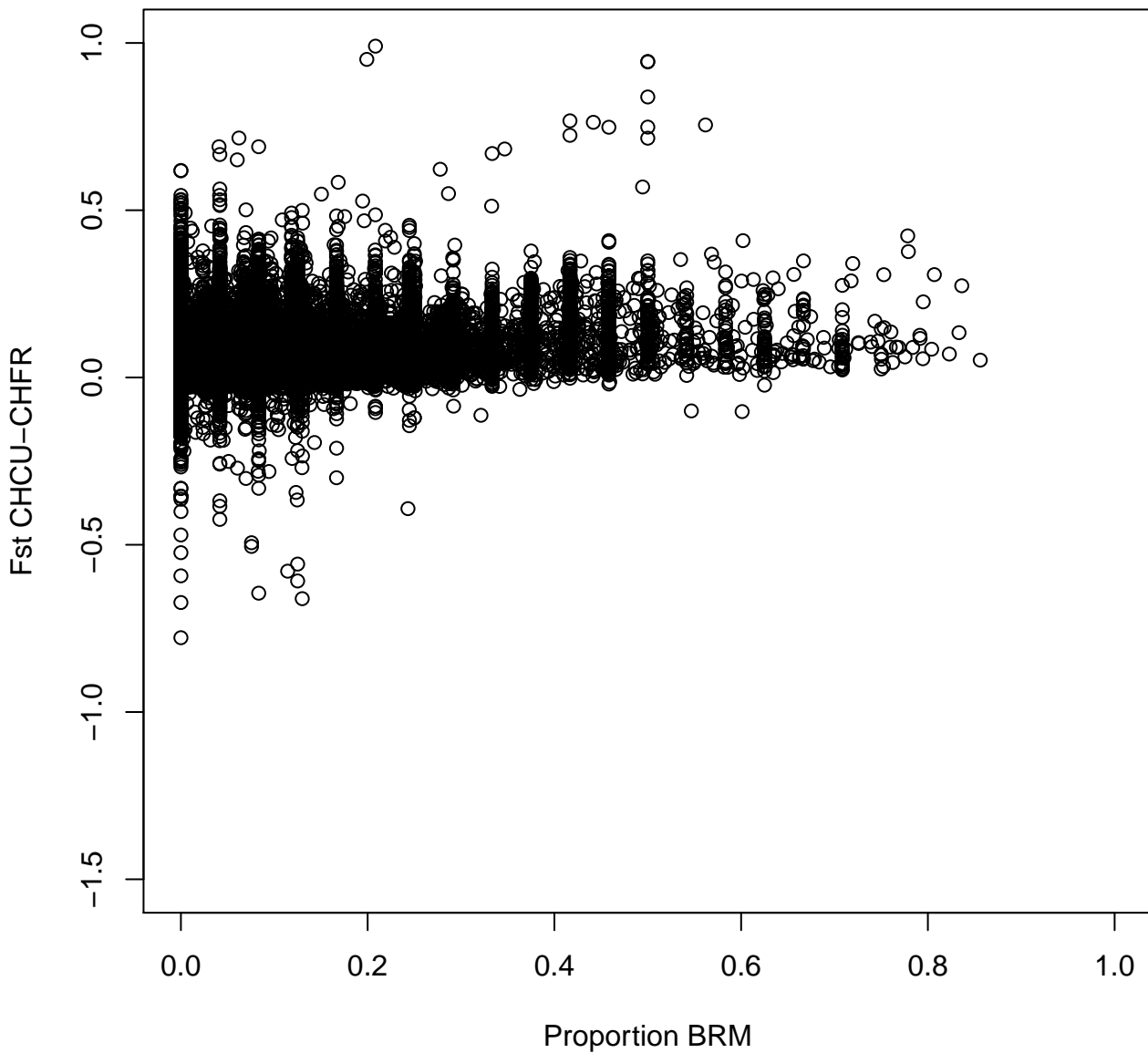

Supplement: Supplementary file 3 — Additional file 3: Figure S2. Relationship between proportion of indicus in CHCU and FST CHCU-CHFR, each dot corresponds to the FST value of a 30-kb window. [file 12711_2020_597_MOESM3_ESM.pdf]
